# Application of ureterorenoscope and flexible ureterorenoscope lithotripsy in removing calculus from extracorporeal living donor renal graft: a single-center experience

**DOI:** 10.1080/0886022X.2017.1349674

**Published:** 2017-07-20

**Authors:** Chun-hua Lin, Zuo-fu Zhang, Jiahui Wang, Lu-xin Yu, Wen-ting Wang, Lei Shi, Xiang-Nan Lin

**Affiliations:** The Affiliated Yantai Yuhuangding Hospital of Qingdao University, Yantai, Shandong, China

**Keywords:** Ureterorenoscope, holmium laser lithotripsy, extracorporeal bench surgery, living donor renal graft, renal calculus

## Abstract

Here, we reported our clinical application of ureterorenoscope (URS) and flexible URS lithotripsy in stone removal on 10 cases of excised living donor kidney graft. After the extraction of donor kidney by retroperitoneal laparoscopy, the donor graft was perfused with 4 °C HCA solution. Calculus between 2–4 mm were removed intact with lithotomy forceps under direct vision of URS. Larger calculi of >4 mm were fractured with flexible URS combining holmium laser lithotripsy. Fragments of the calculus were extracted with basket extractor and lithotomy forceps. All operations were successful. The operation time was 14–31 min (average 21.2 ± 6.3 min). The kidneys were then transplanted to the recipients using routine procedure. The transplanted kidneys functioned well after transplantation. Gross hematuria resolved 1–4 d after operation (average 2.6 ± 0.9 d). The transplanted kidneys functioned well without early complications such as functional recovery delay and acute graft rejection. The donors and recipients were followed for 12 months. The size of the transplanted kidneys was normal and new stones or urinary obstruction was not seen upon urinary color Doppler ultrasound examination. In conclusion, we believe it is feasible, safe and effective to use URS or flexible URS combining holmium laser lithotripsy on extracorporeal living donor kidney.

## Introduction

The limited availability of donor kidneys has made living-relative kidney donation to be one of the main source of renal graft [1]. The presence of renal calculi in the renal graft might sabotage the function restoration of the transplanted kidney due to its potential risk in causing urinary tract obstruction. Therefore, renal calculi used to be considered a clear contraindication for living-relative kidney donation. With the development of laparoscopic surgery and extracorporeal surgery, it is now possible to remove the kidney stones from donor renal grafts by minimally invasive approach, which effectively extended the sources of donor kidneys and promoted the development of living donor transplantation. Here, we report our successful experience of treating 10 cases of kidney stones in extracted renal graft using ureterorenoscope (URS) and flexible URS combined with holmium laser lithotripsy.

## Patients and methods

### Patients

**Figure 1.**
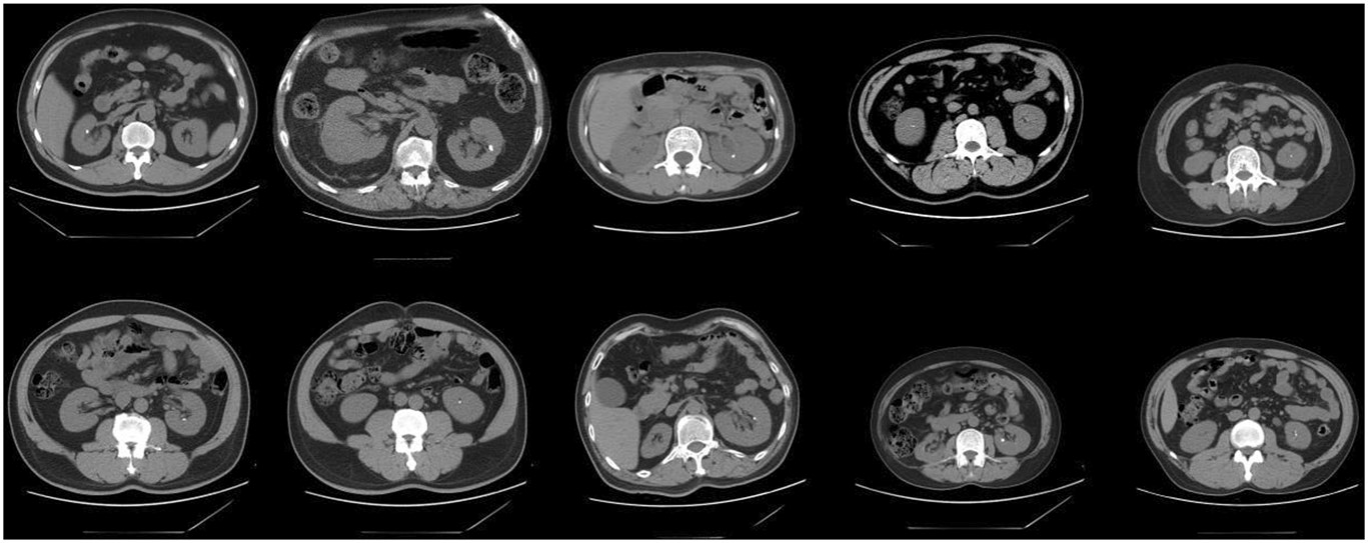
CT images of renal calculi in 10 donor kidneys.

Between July, 2010 and July, 2015, 832 cases of living donor kidney transplantation were performed in our hospital. During the preoperative spiral CT examination, multiple cases exhibited urinary tract calculi, among which 10 cases were unilateral renal calculi (Figure 1). Nine of the 10 cases had solitary renal calculi, and one case had two renal calculi. The average size of stones was 5.8 mm (range between 2 and 9 mm). The calculus was located in the upper calyx in one case, in the middle calyx in three cases and in the lower calyx in six cases. Four donors were male and six were female, aged between 28–58 years old (average 48.8). Eight cases were kidney donation from parent to offspring and two cases were transplantation between siblings. Donor’s renal function was evaluated by laboratory examination, ultrasound, intravenous pyelogram, radioisotope renogram and emission computed tomography (ECT). The donor’s double kidney glomerular filtration rate (GFR) should be >80%, GFR of the donor’s remaining kidney should be ≥40%. In one case, the recipient’s human leucocyte antigen (HLA) was fully matched. In 2, 3, 3 and 1 cases there were 1, 2, 3 and 4 HLA haploid mismatches, respectively. The ABO blood types of the donor and recipient were identical in eight cases and were compatible in the other two cases. All cases were negative in lymphocyte cytotoxicity. Routine preoperative examinations were carried out and no surgical contraindication was identified. The general information and perioperative condition of 10 donors and 10 recipients are summarized in Tables 1 and 2.

**Table 1.** General information and perioperative parameters of 10 donors.

Patient No.	Age/Gender	Blood type	Stone size (mm)	Stone location	Relationship to the recipient	Presurgery Scr (μmol/L)	GFR of donated kidney (mL/min)	GFR of remaining kidney (mL/min)	Surgical procedure	Operation time (min)
1	54/F	A	3	Left UP	Mother	65	50	49	URS + forceps	15
2	48/F	A	2	Left IP	Mother	57	57	53	URS + forceps	14
3	51/F	O	5 + 3	Left LP	Mother	51	51	62	Flexible URS + laser + basket	31
4	58/F	AB	5	Left IP	Mother	48	55	54	Flexible URS + laser + basket	20
5	29M	AB	6	Left LP	sister	55	57	54	Flexible URS + laser + basket	21
6	57/M	O	8	Left IP	Father	62	53	51	Flexible URS + laser + basket	29
7	35/F	AB	9	Left LP	Mother	61	58	52	Flexible URS + laser + basket	28
8	54/M	AB	4	Left LP	Father	57	57	55	URS + forceps	14
9	50/F	A	7	Left LP	Mother	49	52	50	Flexible URS + laser + basket	21
10	52/M	B	6	Left LP	Father	60	53	51	Flexible URS + laser + basket	19

IP: interpolar; LP: lower pole; UP: upper pole; URS: ureterorenoscopy.

**Table 2.** General information and perioperative parameters of the 10 recipients.

Patient No.	Age/Gender	Blood type	HLA mismatch	Protopathy	Pre-surgery Scr (μmol/L)	Complication	Recession time of gross hematuria (d)
1	30/M	A	0	Chronic glomerulonephritis	1500	Nil	1.0
2	29/F	A	3	Chronic glomerulonephritis	1300	Nil	2.5
3	26/M	O	2	Chronic glomerulonephritis	890	Nil	3.0
4	35/M	AB	0	Chronic glomerulonephritis	910	Nil	2.0
5	36/F	O	1	Chronic glomerulonephritis	1056	Nil	3.0
6	31/M	B	3	Chronic glomerulonephritis	1120	Nil	3.5
7	27/M	B	2	Chronic glomerulonephritis	1032	Nil	1.5
8	29/M	O	4	Chronic glomerulonephritis	981	Nil	2.0
9	28/F	A	2	Chronic glomerulonephritis	816	Nil	4.0
10	27/M	B	3	Chronic glomerulonephritis	799	Nil	3.0

### Procedures

**Figure 2.**
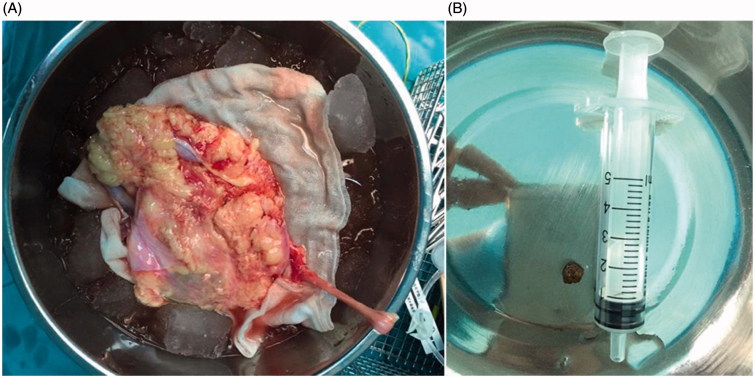
(A) The extracted donor kidney. (B) A 4 mm intact renal calculi removed from a donor kidney.

In all 10 cases, the donor renal graft was obtained via retroperitoneal laparoscopic nephrectomy. The renal graft was placed on sterile gauze after exaction and placed in sterile ice brine (Figure 2(A)). The renal artery was quickly perfused with 4 °C HCA solution at 20–30 cm H_2_O pressure until the kidney turned pale and the drainage from the kidney vein was clear. The renal graft was tailored routinely and the renal artery and vein were sharp or blunt separated, respectively, to ensure enough length for vascular anastomosis. The adipose tissue at the renal hilum was ligatured at about 2 cm from the hilum. While tailoring the ureter, the tissues surrounding the ureter were maintained as much as possible to avoid disruption of the ureteral blood supply. During the operation, we observed that the end of the donor kidney ureter was very narrow; therefore, we attached two 3–0 suture lines to the connective tissue next to the end of ureter for easy traction. An oblique cut was made at the distal end of the ureter. The operation assistant lifted the suture line at the distal end of the ureter to expose the ureterostoma. The 6.5 F/8.5 F URS was retro-inserted through the oblique cut of ureter under low-pressure perfusion. During the insertion of URS, the position of donor graft was adjusted to adapt to the angle of URS to shorten operation time. The calculus of 2–4 mm with smooth edges was extracted intact with lithotomy forceps under direct view from the URS (Figure 2(B)). Calculus of >4 mm were smashed to 2 mm sized pieces using URS combining Koen holmium laser lithotripter and extracted with basket extractor. After all calculi were confirmed to be removed, renal transplantation was performed under routine procedure. All recipients received cyclosporin/mycophenolate mofetil/prednison immunosuppression treatment after transplantation.

### Consent

Written consent and voluntary kidney donation agreement were signed by all donors, recipients and/or their relatives. Transplantations were approved by the Human Organ Transplantation Ethics Committee of Yantai Yuhuangding Hospital. Informed consent was obtained from all participants for using clinical data for scientific research and publication.

## Results

The calculi in all 10 donor renal grafts were successfully removed. The operation time was 14–31 min, average 21.2 ± 6.3 min. There was no damage of the renal capsule, pelvis or ureteral mucosa. One to four days postoperation (average 2.6 ± 0.9 d) gross hematuria disappeared. The transplanted kidneys functioned well without early complications such as functional recovery delay and acute graft rejection. The donors and recipients were followed for 12 months. The size of the transplanted kidneys was normal and new stones or urinary obstruction was not seen upon urinary color Doppler ultrasound examination.

## Discussion

In kidney transplantation, many factors affect the quality of donor kidney and its post-transplantation functional recovery, including age, blood pressure, blood sugar, weight and the presence of renal calculus [2]. Renal calculus used to be an absolute contraindication for live donor kidney transplantation. However, nowadays for the commonly accepted international procedure for donor kidney selection, kidney stones not more than 2 in number, ≤15 mm, on unilateral side with no clinical manifestation are acceptable [2–4]. With the development of auxiliary examination equipment, the detection rate for asymptomatic calculus in donor kidneys has also significantly increased [5].

The treatment method for donor kidney calculus greatly affects the functional recovery of the transplanted kidney. Currently, there is no standard guideline for the treatment of donor kidney calculus, and various routes have been adopted by the transplantation surgeons. Beckly et al. [6] suggested that extracorporeal shock wave lithotripsy (ESWL) could be performed on donor before transplantation, while Trivedi [7] and Devasia [8] confirmed the efficacy of ESWL after transplantation. Rashid [9], Janczak [10] and Vasdev [11] reported extracting calculi from excised donor kidney using URS, flexible URS and pyelolithotomy, respectively, all of which achieved satisfying outcome. The feasibility of URS lithotripsy and percutaneous nephrolithotomy (PCNL) on donor kidney graft after transplantation was confirmed by Benooit [12] and Krambeck [13]. In addition, Devasia [8] and Martin [14] believed that living donor graft containing stones <4 mm could be transplanted without treatment and the calculi usually would naturally pass.

As a matter of fact, no matter lithotripsy or minimally invasive lithotomy was performed before or after kidney transplantation, patient had to undergo trauma and pain. We do not agree with the conservative treatment in which the transplanted kidney is located in fossa iliaca and receives abdominal pressure from the intestines; therefore, its relative position to ureter is easily changed. Furthermore, the transplanted kidney’s ability to expel stones is compromised by reduced ureteral peristalsis due to the loss of innervation. If the change of position of kidney causes obstruction by the stone, the denervated transplant kidney may not exhibit apparent symptoms of obstruction and nephrocolic, which increases treatment difficulty and may even damage renal function [9].

The rapid development of extracorporeal bench surgery provides us more options for treating renal calculus in living donor kidney graft. The advantages of treating transplant kidney extracorporeally are as follows: (i) abides by the ‘absolute sound’ principle of medical ethics; (ii) ensures the quality of donor kidney by reducing operation difficulty, surgery time and additional damage; (iii) abides by the maximum benefit principle of medical ethics and avoids the long-term risk of calculus in the recipient. Therefore, we are more inclined to the strategy used by Rashid [9] and Janczak [10], which is URS or flexible URS combining holmium laser lithotripsy on extracorporeal donor kidney.

Minor damage to the ureteral mucosa has been previously reported for such procedures [15]. The excised kidney does not have blood perfusion, which gave us a clear vision and helped us to locate the stone more quickly. However, iatrogenic mucosal damage is easily neglected. We suggest that the handling should be gentle and careful. When using the lithotomy forceps, it is important to make sure there is no adhesion with surrounding mucosa. If adhesion exists, the stone should be blunt separated with ureteral catheter before removal. In this study, the calculi were not adhered to surrounding tissues in any of the 10 cases.

In summary, we believe that for treating <15 mm unilateral calculi, it is feasible, safe and effective to use URS lithotripsy or flexible URS combining holmium laser lithotripsy on extracorporeal living donor kidney.
